# Exclusive and Combined Use of Statins and Aspirin and the Risk of Pancreatic Cancer: a Case-Control Study

**DOI:** 10.1038/s41598-017-13430-z

**Published:** 2017-10-12

**Authors:** Livia Archibugi, Matteo Piciucchi, Serena Stigliano, Roberto Valente, Giulia Zerboni, Viola Barucca, Michele Milella, Patrick Maisonneuve, Gianfranco Delle Fave, Gabriele Capurso

**Affiliations:** 1 Digestive and Liver Disease Unit, S. Andrea Hospital, Faculty of Medicine and Psychology, Sapienza University of Rome, Rome, Italy; 2 Medical Oncology Unit, Istituto Nazionale Tumori Regina Elena (IFO), Rome, Italy; 3 Division of Epidemiology and Biostatistics, European Institute of Oncology, Milan, Italy

## Abstract

Data on the association between aspirin and statin use and Pancreatic Ductal AdenoCarcinoma (PDAC) risk are conflicting. These drugs are often co-prescribed, but no studies evaluated the potential combined or confounding effect of the two at the same time. We aimed to investigate the association between aspirin and statin exclusive and combined use and PDAC occurrence. Data on environmental factors, family and medical history were screened in a case-control study. PDAC cases were matched to controls for age and gender. Power calculation performed ahead. Odds ratios (OR) and 95% confidence intervals(CI) were obtained from multivariable logistic regression analysis. In 408 PDAC patients and 816 matched controls, overall statin (OR 0.61; 95%CI,0.43–0.88), but not aspirin use was associated to reduced PDAC risk. Compared to non-users, exclusive statin (OR 0.51; 95%CI,0.32–0.80) and exclusive aspirin users (OR 0.64; 95%CI,0.40–1.01) had reduced PDAC risk. Concomitant statin and aspirin use did not further reduce the risk compared with statin use alone and no interaction was evident. Statin protective association was dose-dependent, and consistent in most subgroups, being stronger in smokers, elderly, obese and non-diabetic patients. The present study suggests that statin use is associated to reduced PDAC risk, supporting a chemopreventive action of statins on PDAC.

## Introduction

Pancreatic ductal adenocarcinoma (PDAC) is the 4^th^ leading cause of cancer-related mortality^1^ and is estimated to become the 2^nd^ within 2030^2^. PDAC 5-year survival rate is only 6%^1^ as only 20% of patients are eligible for surgery^3^, and chemo/radiotherapy marginally improve survival in advanced disease^4^. Screening is advised only for high risk individuals in experimental settings^5^.

Prevention might, therefore, play a key role in terms of modifiable lifestyle risk factors such as smoking and/or alcohol drinking, overweight, obesity and diet^6^. Evidence regarding potential chemopreventive drugs for PDAC is instead limited.

Aspirin is a nonsteroidal anti-inflammatory drug (NSAID), irreversible inhibitor of the cyclooxygenase 1/2, widely prescribed for its anti-inflammatory, antipyretic, analgesic and anti-platelet activities. Its chemopreventive role is well recognized for many cancer types^7^ particularly colorectal cancer (CRC)^8,9^.

Preclinical studies demonstrated that aspirin has a role in inhibiting different signaling pathways involved in the carcinogenesis of several cancers including PDAC, such as mTOR, NFkB and Wnt, resulting in enhanced DNA mismatch repair mechanisms, apoptosis, angiogenesis and tumour progression inhibition^10–12^. However, its role in clinical studies on PDAC is less clear, and the most recent meta-analysis on cohort and case-control studies, showed an overall protective effect of aspirin with an odds ratio (OR) of 0.77, although with high heterogeneity^13^.

Statins are 3-hydroxy-3-methyl-glutaryl-coenzyme A (HMG-CoA) reductase inhibitors commonly prescribed for the treatment of hypercholesterolemia and for cardiovascular primary and secondary prevention^14^. The mevalonate pathway involved in their action has a role on multiple signaling cascades such as Ras, MEK, mTOR, BCL-2 and Rho kinases^15–17^, that play role in carcinogenesis and tumour progression. Statin antineoplastic effect is also related with an immunomodulatory and anti-inflammatory activity and with angiogenesis inhibition^18,19^. Their clinical role as chemopreventive agents is still controversial, although a few meta-analyses showed a risk reduction associated with their use, mostly for gastrointestinal tract tumours^20–22^. The latest meta-analysis on the association between statin use and PDAC risk showed no pooled effect^23^. However, since then, other large case-control studies were published^24,25^, recording a reduced PDAC risk associated with statin use.

As aspirin and statins are often co-prescribed, mostly for cardiovascular disease prevention or treatment, one might hypothesize that in studies investigating aspirin, at least a part of the observed effect might be due to the use of statins and *vice versa*. An investigation on the use of both these drugs, alone and in combination, has been conducted by Hoffmeister *et al*. in a case-control study on CRC. The authors reported a stronger risk reduction for statin exclusive use than for that of aspirin, while the combination of the two drugs did not further increase the effect of statins, unless for prolonged uses^26^.

As studies on the possible chemopreventive activity of both aspirin and statins on PDAC gathered heterogeneous results, the analysis of their potential combined or confounding effect in this tumour type seems particularly important. However, there are no studies examining the association of both these two drugs at the same time with PDAC risk.

Therefore, the primary aim of the study was to examine the association between overall and exclusive aspirin and statin use and their combined use with the risk of PDAC. In addition, we conducted subanalyses to explore whether the association was stronger in specific subgroups.

## Materials and Methods

### Study design and population

A single-center case-control study was conducted from January 2006 to February 2016. Patients enrolled in either group provided written informed consent for interviews. The study received local IRB approval at Sant’Andrea Hospital. Methods were performed in accordance with the relevant guidelines and regulations.

Incident cases were prospectively recruited at the gastroenterology department and had PDAC histological diagnosis. Controls consisted of hospital non-patient visitors not genetically related to cases, as well as outpatients and inpatients from the gastroenterology department. Both cases and controls demonstrated the will and ability to participate providing personal data, clinical and cancer history. Exclusion criteria for controls in order to reduce the risk of recruitment bias were: (a) personal history of inflammatory bowel disease, chronic kidney disease or liver cirrhosis, (b) referral to our center for family history (FH) of gastrointestinal cancer, (c) referral to our center or hospitalization for NSAID-induced ulcer disease, (d) personal history of neoplasia in the last 5 years.

We used a frequency matching oversampling controls with a 2:1 ratio. For each case enrolled, the first two eligible controls of the same sex and age (±2 years) were enrolled and interviewed within 30 days.

### Data collection and exposure definition

Data were recorded on a standardized form by a trained physician through direct patient interview; no proxies were interviewed.

The following clinical, epidemiological, therapeutic and morphological parameters were collected: sex, age, race, tobacco and alcohol intake, body mass index (BMI), FH of cancer, previous pancreatic diseases, history of diabetes, aspirin and statin use, length, type and dosage of their use. During the interview, a list of brand and generic medication names for aspirin and statins was provided to help facilitate recall. All cases were interviewed within 1 month from diagnosis and data pertaining the disease were also recorded.

For ever smokers, a consumption of at least 100 cigarettes or >6 months of smoking were needed to be considered a smoker. The total amount of smoking was evaluated as pack-years, defined as the product of packs smoked per day and the total years of smoking. A cut-off of 20 pack-years was set to define heavy smokers. Current smokers were considered as smokers who were currently smoking or who had quit less than 1 year in the past. Ex-smokers were classified as smokers who had quit at least 1 year before the diagnosis of the disease, or its first presentation symptom for cases, or the time of interview for controls.

For ever drinkers, a consumption of at least 12.5 g (1 unit) of alcohol/month was needed to be considered a drinker. One glass of wine, 1 pint or can of beer, one shot of hard liquor were each considered approximatively equal to 12.5 g of alcohol. A cut-off of 21 units/week (262.5 g of alcohol) was set to define heavy drinkers.

This cut-off was used because in a meta-analysis of 156 studies, drinking was reported as a risk factor for many cancers at a dose of both 25 and 50 g/day, and population studies in Italy suggest that 94% of subjects report either being teetotallers or consuming ≤4 alcoholic drinks daily^27,28^.

BMI was calculated as usual adult weight/height^2^ (kg/m^2^) with obesity considered as BMI >30 kg/m^2^. Diabetes was recorded as a potential risk factor when diagnosed >1 year before the diagnosis of cancer or its first symptoms for cases or before the interview for controls.

Aspirin or statin use was defined as the ever use of the medication for at least three consecutive months.

Aspirin users were categorized in “high dosage users” (≥300 mg) and “low dosage users” (≤160 mg)^29^. Statin users were categorized in “low-dosage users” (<20 mg) and “moderate/high dosage users” (≥20 mg) based on median value.

Aspirin or statin users were categorized into different length of duration (<60 months and ≥60 months for aspirin, <48 months and ≥48 months for statins) based on median value.

To avoid possible bias due to cancer symptoms (i.e., weight loss, etc.) or subsequent cancer therapies, subjects were asked about risk factors that were present 12 months before diagnosis or presentation symptoms.

### Statistical analysis

A power calculation was performed ahead: considering an exposure of ~20% for aspirin or statins as previously recorded in 200 controls, to have a 80% power of identifying an odds ratio (OR) ≤ 0.62 as single effect of aspirin or statins, 395 cases and 790 controls were needed. This number would also allow to detect an OR ≤ 0.50 for the combined used of aspirin and statins, based on a 10% combined exposure among controls.

A descriptive analysis was conducted to show the characteristics of PDAC patients at time of diagnosis. Case-control comparisons were made using Chi-square and Fisher’s exact tests where appropriate, for categorical variables and Student’s t-test for continuous variables.

Logistic regression was used to calculate ORs and their 95% confidence intervals (CI). Multivariable logistic regression models were adjusted for potential confounders: age at diagnosis (for cases) or interview (for controls) (5-year age groups), gender, BMI, smoking history, drinking habits, diabetes history, chronic pancreatitis history, FH of PDAC.

All statistical analysis were performed using MedCalc version 13 (MedCalc Software, Belgium). All reported *P* values are 2-sided. *P* values < 0.05 were considered statistically significant.

The STrengthening the Reporting of OBservational studies in Epidemiology (STROBE) checklist for case-control studies was checked for items that should be included in the report.

## Results

Of 421 PDAC cases seen at the Gastroenterology Department during the study period, 9 (2.1%; 4 males and 5 females, mean age 74) were not histologically confirmed. Of the remaining 412, 2 (0.5%) refused to participate and 2 (0.5%) were too ill to take part; of the 893 controls recruited in the same timeframe, 51 (5.7%) were excluded for matching exclusion criteria, 8 (0.9%) refused to be interviewed for privacy concerns, 18 (2.0%) were too ill to take part. This led to a participation rate of 99% for cases and 91% for controls; the analysis was therefore conducted on a final population of 408 cases and 816 matched controls. Respectively 48.7%, 13.4% and 37.9% of the controls, consisted of visitors, inpatients and outpatients. Most of the inpatients were hospitalized for diverticulitis or gastrointestinal infections; outpatients were visiting for either gastro-esophageal reflux disease, irritable bowel syndrome, chronic constipation or dyspepsia.

The median age of cases and matched controls was 68 years (range 35 to 99); 51.2% were men.

Almost all cases and controls were Caucasians.

### Risk factors for pancreatic cancer

Compared with controls, cases had higher mean BMI value, higher proportion of obesity, 1^st^ degree FH of PDAC, previous history of diabetes, previous chronic pancreatitis, and were more frequently smokers, heavy smokers and heavy drinkers (see Table 1).

**Table 1:** Characteristics of pancreatic cancer cases and controls by selected variables of family history, chronic conditions, and lifestyle.

	Cases (408)	Controls (816)	Age and sex adjusted^1^ OR (95% CI)	*P* value	Multivariable analysis^2^ OR (95% CI)	*P* value
Age	68.1 ± 11.6	67.9 ± 11.9	1.00 (0.99–1.01)	0.67	1.00 (0.99–1.02)	0.48
Male gender	209 (51.2%)	418 (51.2%)	1.00 (0.79–1.27)	0.99	0.77 (0.54–1.10)	0.16
1^st^ degree FH of any cancer	218 (53.4%)	403 (49.4%)	1.22 (0.95–1.57)	0.12	—	
2^nd^ degree FH of any cancer	30 (7.4%)	54 (6.6%)	1.29 (0.79–2.11)	0.31	—	
1^st^ degree FH of PDAC	32 (7.8%)	23 (2.8%)	3.02 (1.73–5.26)	0.0001	3.26 (1.79–5.92)	0.0001
2^nd^ degree FH of PDAC	5 (1.2%)	6 (0.7%)	1.83 (0.55–6.09)	0.33	2.17 (0.59–7.97)	0.24
BMI (mean ± Std.dev.)	26.8 ± 4.9	25.9 ± 4.1	1.05 (1.02–1.08)	0.001	1.04 (1.01–1.08)	0.009
BMI >30	79 (19.4%)	120 (14.7%)	1.47 (1.07–2.02)	0.02	—	
History of diabetes	72 (17.6%)	79 (9.7%)	2.03 (1.43–2.88)	<0.0001	1.84 (1.25–2.71)	0.002
Chronic pancreatitis	15 (3.7%)	2 (0.2%)	15.9 (3.60–70.0)	<0.0001	14.7 (3.18–67.6)	0.0006
**Cigarette smoking** ^**3**^						
Never smoker	155 (38.0%)	416 (51.0%)	1.00		1.00	
Ever smoker	253 (62.0%)	400 (49.0%)	1.80 (1.39–2.32)	<0.0001	—	
<20 Pack-years	75 (18.4%)	171 (21.0%)	1.24 (0.89–1.74)	0.21	1.27 (0.88–1.84)	0.19
≥20 Pack-years	153 (37.5%)	229 (28.1%)	1.92 (1.43–2.57)	0.0001	1.93 (1.40–2.66)	<0.0001
**Alcohol drinking** ^**4,5**^						
Never drinker	203 (49.8%)	446 (54.7%)	1.00		1.00	
Ever drinker	173 (42.4%)	370 (45.3%)	0.99 (0.77–1.29)	0.96	—	
<21 alcohol units/week	116 (28.4%)	312 (38.2%)	0.81 (0.61–1.07)	0.14	0.90 (0.66–1.21)	0.47
≥21 alcohol units/week	49 (12.0%)	57 (7.0%)	1.81 (1.17–2.80)	0.008	1.55 (0.96–2.49)	0.07

FH: Family History, PDAC: Pancreatic Ductal AdenoCarcinoma, BMI: Body Mass Index, OR: Odds Ratio, CI: Confidence Intervals.

^1^Odds Ratios adjusted for age (5-year age groups) and gender.

^2^Odds ratios adjusted for age (5-year age groups), sex, body mass index (continuous scale), family history of pancreatic cancer (first and second degree relatives), history of chronic pancreatitis, history of diabetes >1 year, smoking and drinking habits.

^3^Exact amount and duration not recalled by 25 (6.1%) cases and 0 controls.

^4^Exact amount and duration not recalled by 32 (7.8%) cases and 0 controls.

^5^Unknown units/week for 8 (2.0%) cases and 1 (0.1%) control.

### Overall use of aspirin and statins and risk of pancreatic cancer

Seventy-eight cases (19.1%) and 191 controls (23.4%) reported ever use of aspirin, which was associated with a statistically borderline significant reduced PDAC risk (age- and sex-adjusted OR, 0.74; 95% CI, 0.54–1.02; Table 2). Only 2 of the 78 cases and 5 of the 191 controls (0.5% of both cases and controls) were on high-dose aspirin. The median duration of aspirin use was 60 months for both cases and controls, with 20 patients (4.9%) among cases and 33 patients (4.0%) among controls not recalling the exact duration of use. A borderline significant protective association was recorded for shorter duration of exposure.

**Table 2:** Overall aspirin and statin use among pancreatic cancer cases and controls.

	Cases (408)	Controls (816)	Age and sex adjusted^1^ OR (95% CI)	*P* value	Multivariable analysis^2^ OR (95% CI)	*P* value
**Aspirin use** ^3,4^						
Never	330 (80.9%)	625 (76.6%)	1.00			
Ever	78 (19.1%)	191 (23.4%)	0.74 (0.54–1.02)	0.06	0.77 (0.53–1.11)	0.16
Low-dose (≤160 mg)	68 (16.7%)	154 (18.9%)	0.80 (0.58–1.12)	0.20		
High-dose (≥300 mg)	2 (0.5%)	5 (0.6%)	0.72 (0.14–3.76)	0.70		
<60 months	25 (6.1%)	73 (9.0%)	0.62 (0.38–1.01)	0.05		
≥60 months	33 (8.1%)	85 (10.4%)	0.70 (0.45–1.09)	0.11		
**Statin use** ^5–7^						
Never	334 (81.9%)	613 (75.1%)	1.00			
Ever	74 (18.1%)	203 (24.9%)	0.64 (0.48–0.88)	0.005	0.61 (0.43–0.88)	0.007
Atorvastatin	29 (7.1%)	85 (10.4%)	0.60 (0.38–0.94)	0.03		
Simvastatin	23 (5.6%)	45 (5.5%)	0.92 (0.54–1.56)	0.76		
Other forms*	11 (2.7%)	27 (3.3%)	0.72 (0.35–1.48)	0.37		
<20 mg	14 (3.4%)	44 (5.4%)	0.56 (0.30–1.05)	0.07		
≥20 mg	22 (5.4%)	90 (11.0%)	0.43 (0.27–0.71)	0.0008		
<48 months	25 (6.1%)	84 (10.3%)	0.53 (0.33–0.84)	0.008		
≥48 months	22 (5.4%)	71 (8.7%)	0.55 (0.33–0.90)	0.02		

OR: Odds Ratio, CI: Confidence Interval.

*Fluvastatin, Lovastatin, Pravastatin, Rosuvastatin.

^1^Odds Ratios adjusted for age (5-year age groups) and gender.

^2^Odds ratios adjusted for age (5-year age groups), sex, body mass index (continuous scale), family history of pancreatic cancer (first and second degree relatives), history of chronic pancreatitis, history of diabetes >1 year, smoking and drinking habits.

^3^Unknown dose for 8 (2%) cases and 32 (3.9%) controls.

^4^Unknown duration for 20 (4.9%) cases and 33 (4%) controls.

^5^Unknown type for 11 (2.7%) cases and 46 (5.6%) controls.

^6^Unknown dose for 38 (9.3%) cases and 69 (8.5%) controls.

^7^Unknown duration for 27 (6.6%) cases and 48 (5.9%) controls.

Seventy-four cases (18.1%) and 203 controls (24.9%) reported ever use of statins, which was associated with a statistically significant reduced PDAC risk (age- and sex-adjusted OR, 0.64; 95% CI, 0.48–0.88; Table 2). The median dosage of statins was 20 mg and the median duration of use was 48 months for both cases and controls. A higher dosage of statins (for ≥20 mg, OR, 0.43; 95% CI, 0.27–0.71) was associated with a stronger protective effect. The most commonly used statin was atorvastatin. Of the 74 cases and the 203 controls, respectively 11 (2.7%) and 46 (5.6%) could not recall the type of drug, 38 (9.3%) and 69 (8.5%) could not recall the dosage, and 27 (6.6%) and 48 (5.9%) could not recall the duration of use.

At multivariable analysis, statins (OR 0.61; 95% CI, 0.43–0.88) but not aspirin (OR, 0.77; 95% CI, 0.53–1.11) use was associated to a reduced PDAC risk (Table 2). OR and 95% CI for all other risk factors for this first multivariable analysis model are reported in Supplementary Table 1.

In order to avoid possible bias due to controls selection, a sensitive analysis for control type (visitors or hospital patients) was performed. At multivariable analysis, statin use was associated to a reduced risk of PDAC both with sensitive analysis restricted to visitors (OR 0.60; 95% CI, 0.40–0.89) or to hospital controls (OR 0.59; 95% CI 0.40–0.87), while the use of aspirin was not associated to PDAC risk for either visitors (OR, 0.82; 95% CI, 0.55–1.23) nor hospital controls (OR 0.67; 95% CI 0.45–1.01).

### Exclusive and Combined use of Aspirin and Statins and risk of pancreatic cancer

In order to evaluate the exclusive or combined effect of the two drugs and avoid possible confounding effects, we analyzed the use of aspirin excluding patients reporting also the use of statins and *vice versa* (Table 3). An exclusive aspirin use was recorded in 39 (9.6%) of all cases and 95 (11.6%) of all controls, and an exclusive statin use was recorded in 35 (8.6%) of cases and 107 (13.1%) of controls. In an age- and sex- adjusted analysis the exclusive use of statins was associated with a stronger risk reduction (OR, 0.54; 95% CI, 0.36–0.82) than the exclusive use of aspirin (OR, 0.67; 95% CI, 0.45–1.02). The concomitant use of statins and aspirin was reported in 39 cases (9.6%) and 96 controls (11.8%). This combined use did not further reduce the risk (OR, 0.67; 95% CI, 0.44–1.01) compared with the use of statins alone.

**Table 3:** Exclusive and combined aspirin and statin use among pancreatic cancer cases and controls.

	Cases (408)	Controls (816)	Age and sex adjusted^1^ OR (95% CI)	*P* value	Multivariable analysis^2^ OR (95% CI)	*P* value
**Exclusive or combined use**						
Neither aspirin nor statins	295 (72.3%)	518 (63.5%)	1.00		1.00	
Aspirin only	39 (9.6%)	95 (11.6%)	0.67 (0.45–1.02)	0.06	0.64 (0.40–1.01)	0.06
Statins only	35 (8.6%)	107 (13.1%)	0.54 (0.36–0.82)	0.004	0.51 (0.32–0.80)	0.004
Aspirin and Statins	39 (9.6%)	96 (11.8%)	0.67 (0.44–1.01)	0.06	0.54 (0.34–0.87)	0.01

OR: Odds Ratio, CI: Confidence Interval.

^1^Odds Ratios adjusted for age (5-year age groups) and gender.

^2^Odds ratios adjusted for age (5-year age groups), sex, body mass index (continuous scale), family history of pancreatic cancer (first and second degree relatives), history of chronic pancreatitis, history of diabetes >1 year, smoking and drinking habits.

At multivariable analysis adjusted for other potential confounding factors, statin use (OR, 0.51; 95% CI, 0.32–0.80) was associated to a reduced risk of PDAC occurrence, while the association for aspirin use was of borderline significance (OR, 0.64; 95% CI, 0.40–1.01). Also at multivariable analysis the combined use of the two drugs did no further reduce the risk compared to the use of statins alone (OR, 0.54; 95% CI, 0.34–0.87). We found no evidence of interaction between statin and aspirin (P = 0.17), when adding an interaction term to the main effects of aspirin and statin in a multivariable model adjusted for other potential confounding variables (Supplementary Table 2).

### Association between use of aspirin and statins and risk of pancreatic cancer in subgroups

**Figure 1:**
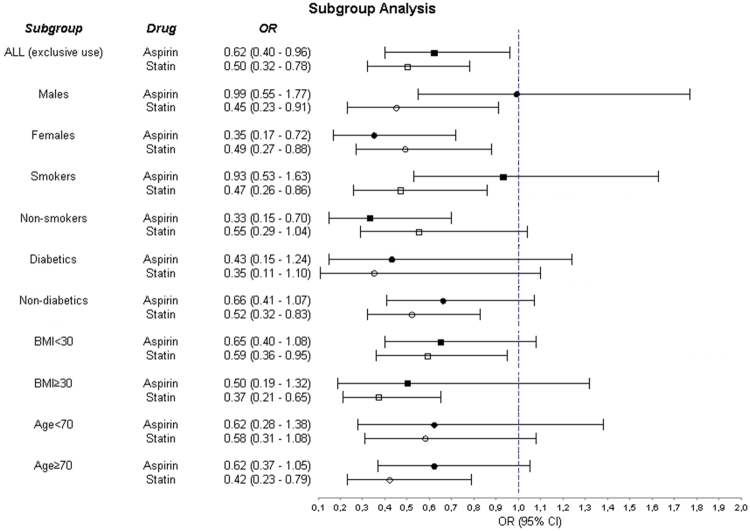
Subgroup analysis of the association between the exclusive use of either statins or aspirin and pancreatic cancer risk. Subgroup estimates are adjusted for age, sex, body mass index, first degree family history of pancreatic cancer, history of diabetes >1 year and smoking habit. OR: Odds Ratio, CI: Confidence Interval.

To evaluate a potential specific association between aspirin and statin use and the risk of PDAC among different subgroups, separate multivariable subgroup analyses were conducted for the exclusive use of the two drugs according to: gender, smoking habit, obesity, history of diabetes and age ≥ or <70 years. Results are shown in Fig. 1.

## Discussion

This is the first study evaluating the possible association between overall, exclusive and combined use of both aspirin and statins and PDAC risk at the same time. This is a relevant issue, as both drugs together are very frequently prescribed in elderlies for cardiovascular prevention and treatment^30^.

Therefore, one might hypothesize that both a confounding effect, for which only one of the two co-prescribed drugs is protective, or a synergistic effect, for which the activity of the combination of the two drugs is superior to their exclusive use, might occur.

In the present study, PDAC risk is inversely associated with the overall statin use, with a dosage-dependent effect. The overall aspirin use is not associated to a statistically significant reduced PDAC risk at multivariable analysis (Table 2). Both exclusive aspirin and statin use were related to a reduced PDAC risk, with statin use showing a risk reduction of 49%, higher than that of aspirin, which was only borderline statistically significant. The concomitant use of the two drugs was also associated to a 46% reduced PDAC risk, without conferring a stronger risk reduction compared to the use of statins alone, therefore we might speculate that the main protective effect of the combined use is due to statins. Furthermore, the analysis of interaction did not suggest a synergistic effect (Supplementary Table 2).

A protective association with the use of statins has been described mostly in case-control studies and not in cohort studies before. Two recent cohort^31^ and case-control studies^32^ evaluated the use of statins and the risk of PDAC. The first study on a female population adjusted the analysis for the use of aspirin and NSAIDs, and the second also analysed the use of aspirin, but both did not evaluate the association of the two drugs or their exclusive use. Two other large case-control studies^24,25^ evaluating the association between statin use and PDAC risk instead showed a risk reduction similar to that of the present study. However, in none of those previous publications, *a priori* power calculation was performed. Walker *et al*.^24^ reported a reduced PDAC risk in overall statin users with OR = 0.66 and, in a subgroup analysis only in men and mostly for prolonged use. Carey *et al*.^25^, described a reduced risk only in male smokers. However, in this latter study, OR were not adjusted for BMI and dosage and duration of drug use were not recorded. Compared to those previous studies, our results show a more consistent association between the exclusive use of statins and PDAC risk reduction, which was similar for both genders, but limited to smokers, elderly subjects, obese and non-diabetic patients (Fig. 1). Statins might exert a specific protective effect on cigarette-related carcinogenesis, as they have also been found to be protective against lung cancer^33^. Moreover, nicotine-mediated pancreatic carcinogenesis in animal models relies on the activation of AKT and ERK^34^, and statins have been reported to negatively regulate such signaling pathways in PDAC models and in other cancers^35,36^. The reasons for a more important protective effect of statins in non-diabetics are unclear. Chen *et al*., evaluated a cohort of diabetic patients, in whom statin use was associated to a reduced risk^37^. A more complex relation between statins and diabetes might be due to their pro-diabetogenic effect^38^. Moreover, although we considered only patients with a history of diabetes >1 year, in order to avoid PDAC causing, rather than being caused by diabetes, one cannot exclude that in some patients the onset of diabetes, although >1 year, might still be due to PDAC occurrence^39^.

As far as regards the more significant association between statin use and reduced PDAC risk in subjects aged ≥70 years, this can be explained by the obvious increased PDAC risk in older subjects. Furthermore, an older age is usually associated to prolonged drug exposure.

There are many published studies on the preventive effect of aspirin on PDAC occurrence, with heterogeneous results^7,40–42^ but, to our knowledge, no significant results were reported for specific subgroups. In our study aspirin was protective only in females and non-smokers. These results are in contrast with those of a previous large cohort study conducted on nurses by Schernhammer *et al*.^41^, where aspirin intake was a risk factor for PDAC, directly related to the number of tablets taken. The results of our multivariable analyses (Tables 2 and 3) and of the subgroup analysis for the exclusive use of the two drugs (Fig. 1), suggest an inconsistent effect of aspirin, possibly supporting the hypothesis that at least a part of the previously reported association between aspirin use and reduced PDAC risk is due to concomitant statin use.

However, as in our population there was a very low proportion of high-dosage aspirin users, we cannot exclude a stronger protective effect for higher dosages of aspirin, although in previous studies also low-dose aspirin showed protective effect^40^.

Among the different statin types, atorvastatin was the most frequently prescribed in the present study, and was associated to a reduced risk of PDAC. The chemopreventive effect of atorvastatin has already been described in *in vivo* studies^35,43^, while previous cohort or case-control studies showed heterogeneous results between different statins^23,24,31^. This could therefore be only due to atorvastatin being the most frequently prescribed type of statins.

The present study has some strengths: 1) it is the first specifically aimed at evaluating the association between overall, exclusive and combined use of both aspirin and statins and PDAC risk, as they both seem to have chemopreventive properties and are very often prescribed together; 2) it is one of the few studies on this topic with an *a priori* power calculation; 3) as expected, an increased PDAC risk for patients with multiple 1^st^ degree FH of neoplasia, 1^st^ degree FH of PDAC, increased BMI, previous history of diabetes, chronic pancreatitis, smoking habit was seen, suggesting the absence of biases and the genuineness of our population; 4) statin results are consistent with most Bradford Hill criteria for causation^44^ as the association is strong, a biological gradient is evident as higher dosages lower the risk and the association is plausible in terms of mechanisms and coherent with *in vitro* studies. A clear temporal relation was instead not evident. Of course, as this is a case-control study, causation of an effect cannot be observed and we can only report an association with risk, useful to generate hypotheses that need validation.

There are, however, some limitations. First, as for any case-control study, the risk of recall bias has to be taken into account, although when interviewed, patients were provided with a list of brand and dosages in order to reduce this risk. Furthermore, both the rate of non-participants and the rate of patients not recalling data about their drug use are similar among cases and controls. In order to reduce the risk of confounding factors, the questionnaire was carefully conducted by a trained physician with expertise on pancreatic disorders, asking information regarding risk factors exposure as present 1 year before the interview. As in any case-control study the choice of the control population is a possible matter of concern. We opted for a control group that we believed to represent the same population as the case group, derived from the same catchment area, and being composed both of patients seen for similar symptoms by the same doctors in the same clinics but with a final diagnosis unrelated with the disease of interest, and by hospital non-patient visitors; this, therefore, was unlikely to cause a specific bias^45^. To explore the possibility of a potential selection bias among hospital controls, who might have been less likely to use aspirin being selected in a Gastroenterology setting, we performed a sensitivity analysis comparing the effect of aspirin and statins in the two different control groups (visitors and hospital controls) separately with findings suggesting a consistent effect of statins but not of aspirin.

Furthermore, our control group seems to be representative of the general Italian population in terms of environmental risk factors and exposure to the drugs of interest^46,47^ and to populations used for other case-control studies on PDAC^48^. Also, as the study was not powered for the exclusive use analyses or subgroup analysis, these results have to be taken carefully into account as they might not be conclusive. For example, in a post-hoc calculation limited to the 653 smokers (253 cases and 400 controls), the statistical power of the study dropped to 67% for detecting an OR = 0.62 (as per protocol) considering the actual aspirin exposure (26%) measured among control smokers.

Furthermore, one cannot exclude that the observed reduced PDAC risk associated with statin use and to a lesser extent of aspirin, is a surrogate for other uninvestigated factors such as a healthier lifestyle. At any rate, the current analyses were corrected for most known risk factors associated with PDAC risk (see Table 1), but residual confounding due to other factors such as diet, antioxidants use, physical activity or different indication for statin use cannot be excluded. Notably, a recent meta-analysis showed no association between serum cholesterol levels and PDAC risk^49^.

The biological mechanisms through which statins might prevent PDAC are not completely clarified. Interestingly, the importance of statins has been recently proved in retrospective studies also in terms of prolonging survival in operated PDAC patients^50–52^, suggesting again an effect of this class of drugs on this tumour. In this context, the development of randomized controlled trials investigating the effect of statins in an adjuvant setting, in patients undergoing resection for PDAC could prove interesting. Moreover, as the statistical power of such studies might be limited by the very low rate of survival of PDAC patients, further studies on the possible chemopreventive effect of statins in individuals with an increased risk of developing PDAC, such as patients with genetic syndromes at high risk of PDAC^5^, and patients diagnosed with pancreatic pre-neoplastic lesions such as Intraductal Papillary Mucinous Neoplasia (IPMNs)^53^ might be of interest.

In conclusion, the present study suggests that statin use, rather than that of aspirin, in particular at higher dosages is associated to a reduced PDAC risk. These findings support a chemopreventive action of statins on PDAC and no apparent synergistic activity of the two medications.

## Electronic supplementary material

Supplementary Tables

## References

[1] Siegel R, Naishadham D, Jemal A (2013). Cancer statistics, 2013. CA: a cancer journal for clinicians.

[2] Rahib L (2014). Projecting cancer incidence and deaths to 2030: the unexpected burden of thyroid, liver, and pancreas cancers in the United States. Cancer research.

[3] Spanknebel K, Conlon KC (2001). Advances in the surgical management of pancreatic cancer. Cancer journal.

[4] Neuzillet C (2015). State of the art and future directions of pancreatic ductal adenocarcinoma therapy. Pharmacology & therapeutics.

[5] Canto, M. I. *et al*. Frequent detection of pancreatic lesions in asymptomatic high-risk individuals. *Gastroenterology* **142**, 796–804; quiz e714–795, 10.1053/j.gastro.2012.01.005 (2012). 10.1053/j.gastro.2012.01.005 PMC3321068 22245846

[6] Maisonneuve P, Lowenfels AB (2015). Risk factors for pancreatic cancer: a summary review of meta-analytical studies. International journal of epidemiology.

[7] Cao Y (2016). Population-wide Impact of Long-term Use of Aspirin and the Risk for Cancer. JAMA oncology.

[8] Bibbins-Domingo K, Force USPST (2016). Aspirin Use for the Primary Prevention of Cardiovascular Disease and Colorectal Cancer: U.S. Preventive Services Task Force Recommendation Statement. Annals of internal medicine.

[9] Emilsson L (2017). Systematic review with meta-analysis: the comparative effectiveness of aspirin vs. screening for colorectal cancer prevention. Alimentary pharmacology & therapeutics.

[10] Shen X (2013). Aspirin: a potential therapeutic approach in pancreatic cancer. Current medicinal chemistry.

[11] Din, F. V. *et al*. Aspirin inhibits mTOR signaling, activates AMP-activated protein kinase, and induces autophagy in colorectal cancer cells. *Gastroenterology* **142**, 1504–1515 e1503, 10.1053/j.gastro.2012.02.050 (2012). 10.1053/j.gastro.2012.02.050 PMC3682211 22406476

[12] Yue W, Yang CS, DiPaola RS, Tan XL (2014). Repurposing of metformin and aspirin by targeting AMPK-mTOR and inflammation for pancreatic cancer prevention and treatment. Cancer prevention research.

[13] Zhang YP, Wan YD, Sun YL, Li J, Zhu RT (2015). Aspirin might reduce the incidence of pancreatic cancer: A meta-analysis of observational studies. Scientific reports.

[14] Miller PE, Martin SS (2016). Approach to Statin Use in 2016: an Update. Current atherosclerosis reports.

[15] Laezza C (2010). Inhibition of 3-hydroxy-3-methylglutaryl-coenzyme A reductase activity and of Ras farnesylation mediate antitumor effects of anandamide in human breast cancer cells. Endocrine-related cancer.

[16] Duncan RE, El-Sohemy A, Archer MC (2005). Statins and cancer development. Cancer epidemiology, biomarkers & prevention: a publication of the American Association for Cancer Research, cosponsored by the American Society of Preventive Oncology.

[17] Spampanato C (2012). Simvastatin inhibits cancer cell growth by inducing apoptosis correlated to activation of Bax and down-regulation of BCL-2 gene expression. International journal of oncology.

[18] Demierre MF, Higgins PD, Gruber SB, Hawk E, Lippman SM (2005). Statins and cancer prevention. Nature reviews. Cancer.

[19] Dulak J, Jozkowicz A (2005). Anti-angiogenic and anti-inflammatory effects of statins: relevance to anti-cancer therapy. Current cancer drug targets.

[20] Bonovas S (2014). Statins: do they have a potential role in cancer prevention and modifying cancer-related outcomes?. Drugs.

[21] Liu Y (2014). Association between statin use and colorectal cancer risk: a meta-analysis of 42 studies. Cancer causes & control: CCC.

[22] Alexandre L, Clark AB, Cheong E, Lewis MP, Hart AR (2012). Systematic review: potential preventive effects of statins against oesophageal adenocarcinoma. Alimentary pharmacology & therapeutics.

[23] Cui X (2012). Statin use and risk of pancreatic cancer: a meta-analysis. Cancer causes & control: CCC.

[24] Walker EJ, Ko AH, Holly EA, Bracci PM (2015). Statin use and risk of pancreatic cancer: results from a large, clinic-based case-control study. Cancer.

[25] Carey FJ (2013). The differential effects of statins on the risk of developing pancreatic cancer: a case-control study in two centres in the United Kingdom. Digestive diseases and sciences.

[26] Hoffmeister M, Chang-Claude J, Brenner H (2007). Individual and joint use of statins and low-dose aspirin and risk of colorectal cancer: a population-based case-control study. International journal of cancer.

[27] Corrao G, Bagnardi V, Zambon A, La Vecchia C (2004). A meta-analysis of alcohol consumption and the risk of 15 diseases. Preventive medicine.

[28] Loguercio C (2007). Drinking habits and risk of altered liver enzymes in the general population of a rural area in Southern Italy. Digestive and liver disease: official journal of the Italian Society of Gastroenterology and the Italian Association for the Study of the Liver.

[29] Patrignani P, Patrono C (2016). Aspirin and Cancer. Journal of the American College of Cardiology.

[30] Lafeber, M. *et al*. The combined use of aspirin, a statin, and blood pressure-lowering agents (polypill components) and the risk of vascular morbidity and mortality in patients with coronary artery disease. *American heart journal* **166**, 282–289 e281, 10.1016/j.ahj.2013.04.011 (2013). 10.1016/j.ahj.2013.04.011 23895811

[31] Simon MS (2016). Prospective analysis of association between statins and pancreatic cancer risk in the Women’s Health Initiative. Cancer causes & control: CCC.

[32] Kho PF (2016). Nonsteroidal anti-inflammatory drugs, statins, and pancreatic cancer risk: a population-based case-control study. Cancer causes & control: CCC.

[33] Liu, J. C. *et al*. Statins dose-dependently exert a chemopreventive effect against lung cancer in COPD patients: a population-based cohort study. *Oncotarget*, 10.18632/oncotarget.11162 (2016). 10.18632/oncotarget.11162 PMC5312335 27517752

[34] Hermann, P. C. *et al*. Nicotine promotes initiation and progression of KRAS-induced pancreatic cancer via Gata6-dependent dedifferentiation of acinar cells in mice. *Gastroenterology* **147**, 1119–1133 e1114, 10.1053/j.gastro.2014.08.002 (2014). 10.1053/j.gastro.2014.08.002 25127677

[35] Mohammed A (2012). Atorvastatin delays progression of pancreatic lesions to carcinoma by regulating PI3/AKT signaling in p48Cre/+ LSL-KrasG12D/+ mice. International journal of cancer.

[36] Tsubaki M (2016). Statins inhibited the MIP-1alpha expression via inhibition of Ras/ERK and Ras/Akt pathways in myeloma cells. Biomedicine & pharmacotherapy = Biomedecine & pharmacotherapie.

[37] Chen MJ (2016). Statins and the risk of pancreatic cancer in Type 2 diabetic patients–A population-based cohort study. International journal of cancer.

[38] Backes JM, Kostoff MD, Gibson CA, Ruisinger JF (2016). Statin-Associated Diabetes Mellitus: Review and Clinical Guide. Southern medical journal.

[39] Pannala R, Basu A, Petersen GM, Chari ST (2009). New-onset diabetes: a potential clue to the early diagnosis of pancreatic cancer. The Lancet. Oncology.

[40] Streicher SA, Yu H, Lu L, Kidd MS, Risch HA (2014). Case-control study of aspirin use and risk of pancreatic cancer. Cancer epidemiology, biomarkers & prevention: a publication of the American Association for Cancer Research, cosponsored by the American Society of Preventive Oncology.

[41] Schernhammer ES (2004). A prospective study of aspirin use and the risk of pancreatic cancer in women. Journal of the National Cancer Institute.

[42] Capurso G (2007). Meta-analysis: the use of non-steroidal anti-inflammatory drugs and pancreatic cancer risk for different exposure categories. Alimentary pharmacology & therapeutics.

[43] Liao J (2013). Atorvastatin inhibits pancreatic carcinogenesis and increases survival in LSL-KrasG12D-LSL-Trp53R172H-Pdx1-Cre mice. Molecular carcinogenesis.

[44] Hill AB (1965). The Environment and Disease: Association or Causation?. Proceedings of the Royal Society of Medicine.

[45] Lewallen S, Courtright P (1998). Epidemiology in practice: case-control studies. Community eye health/International Centre for Eye Health.

[46] Ferrajolo C (2014). Pattern of statin use in southern italian primary care: can prescription databases be used for monitoring long-term adherence to the treatment?. PloS one.

[47] (ISTAT), I. N. d. S. http://dati.istat.it/Index.aspx?DataSetCode (Date of access:15/06/2017) (2016).

[48] Hassan MM (2007). Risk factors for pancreatic cancer: case-control study. The American journal of gastroenterology.

[49] Wang J, Wang WJ, Zhai L, Zhang DF (2015). Association of cholesterol with risk of pancreatic cancer: a meta-analysis. World journal of gastroenterology.

[50] Kozak MM (2016). Statin and Metformin Use Prolongs Survival in Patients With Resectable Pancreatic Cancer. Pancreas.

[51] Jeon CY (2015). The association of statin use after cancer diagnosis with survival in pancreatic cancer patients: a SEER-medicare analysis. PloS one.

[52] Wu BU (2015). Impact of statin use on survival in patients undergoing resection for early-stage pancreatic cancer. The American journal of gastroenterology.

[53] Distler M, Aust D, Weitz J, Pilarsky C, Grutzmann R (2014). Precursor lesions for sporadic pancreatic cancer: PanIN, IPMN, and MCN. BioMed research international.

