# Supplementary material for: Exclusive and Combined Use of Statins and Aspirin and the Risk of Pancreatic Cancer: a Case-Control Study
Source: Sci Rep. 2017 Oct 12;7:13024. doi: 10.1038/s41598-017-13430-z (PMC5638859; doi:10.1038/s41598-017-13430-z)
Supplement: Supplementary file 1 — Supplementary Tables [file 41598_2017_13430_MOESM1_ESM.pdf]

Exclusive and Combined Use of Statins and Aspirin and the Risk of Pancreatic Cancer: a Case-Control Study R2: Supplementary Files

Livia Archibugi MD, Matteo Piciucchi MD, Serena Stigliano MD, Roberto Valente MD, Giulia Zerboni MD, Viola Barucca MD, Michele Milella MD, Patrick Maisonneuve Eng, Gianfranco Delle Fave MD, Gabriele Capurso MD PhD

Supplementary Table 1. Full multivariable model for the association between aspirin, statins and PDAC

	**Multivariable analysis OR (95% CI)**	***P* value**
Age	1.01 (1.00-1.02)	0.24
Male gender	0.86 (0.66-1.12)	0.27
1st degree FH of PDAC	3.22 (1.82-5.70)	0.0001
BMI (mean ± Std.dev.)	1.05 (1.02-1.08)	0.002
History of diabetes	2.00 (1.36-2.92)	0.0004
Chronic pancreatitis	17.01 (3.78-76.57)	0.0002
Ever smoker	1.87 (1.43-2.45)	<0.0001
Aspirin use	0.76 (0.54-1.07)	0.12
Statin use	0.60 (0.43-0.84)	0.003

FH: Family History, PDAC: Pancreatic Ductal AdenoCarcinoma, BMI: Body Mass Index, OR: Odds Ratio, CI: Confidence Interval

Supplementary Table 2. Exclusive use of aspirin and statin and their interaction among pancreatic cancer cases and controls

	**Cases (408)**	**Controls (816)**	**Age and sex adjusted[1] OR (95% CI)**	***P* value**	**Multivariable analysis[2] OR (95% CI)**	***P* value**
**Exclusive or combined use**						
Neither aspirin nor statins	295 (72.3%)	518 (63.5%)	1.00		1.00	
Aspirin only	39 (9.6%)	95 (11.6%)	0.67 (0.45-1.02)	0.06	0.64 (0.40-1.01)	0.06
Statins only	35 (8.6%)	107 (13.1%)	0.54 (0.36-0.82)	0.004	0.51 (0.32-0.80)	0.004
Interaction	39 (9.6%)	96 (11.8%)	1.84 (0.94-3.60)	0.08	1.68 (0.80-3.54)	0.17

OR: Odds Ratio, CI: Confidence Interval

[1]Odds Ratios adjusted for age (5-year age groups) and gender

[2]Odds ratios adjusted for age (5-year age groups), sex, body mass index (continuous scale), family history of pancreatic cancer (first and second degree relatives), history of chronic pancreatitis, history of diabetes > 1 year, smoking and drinking habits.
